# The effects of human pregnancy-specific β1-glycoprotein preparation on Th17 polarization of CD4^+^ cells and their cytokine profile

**DOI:** 10.1186/s12865-020-00385-6

**Published:** 2020-10-30

**Authors:** Valeria P. Timganova, Svetlana A. Zamorina, Larisa S. Litvinova, Natalia M. Todosenko, Maria S. Bochkova, Pavel V. Khramtsov, Mikhail B. Rayev

**Affiliations:** 1 Institute of Ecology and Genetics of Microorganisms, Ural Branch of the Russian Academy of Sciences - branch of the Perm Federal Research Center, Ural Branch of the Russian Academy of Sciences, Goleva str., 13, Perm, Russian Federation 614081; 2 Immanuel Kant Baltic Federal University, A. Nevsky str., 14, Kaliningrad, Russian Federation 236016

**Keywords:** Pregnancy-specific β1-glycoprotein, Helper T cells, Pregnancy, Cytokine profile, RORγτ transcription factor, Interleukin-17

## Abstract

**Background:**

Pregnancy-specific β1-glycoproteins are capable of regulating innate and adaptive immunity, exerting predominantly suppressive effects. In this regard, they are of interest in terms of their pharmacological potential for the treatment of autoimmune diseases and post-transplant complications. The effect of these proteins on the main pro-inflammatory subpopulation of T lymphocytes, IL-17-producing helper T cells (Th17), has not been comprehensively studied. Therefore, the effects of the native pregnancy-specific β1-glycoprotein on the proliferation, Th17 polarization and cytokine profile of human CD4^+^ cells were assessed.

**Results:**

Native human pregnancy-specific β1-glycoprotein (PSG) at а concentration of 100 μg/mL was shown to decrease the frequency of Th17 (RORγτ^+^) in CD4^+^ cell culture and to suppress the proliferation of these cells (RORγτ^+^Ki-67^+^), along with the proliferation of other cells (Ki-67^+^) (*n* = 11). A PSG concentration of 10 μg/mL showed similar effect, decreasing the frequency of Ki-67^+^ and RORγτ^+^Ki67^+^ cells. Using Luminex xMAP technology, it was shown that PSG decreased IL-4, IL-5, IL-8, IL-12, IL-13, IL-17, MIP-1β, IL-10, IFN-γ, TNF-α, G-CSF, and GM-CSF concentrations in Th17-polarized CD4^+^ cell cultures but did not affect IL-2, IL-7, and MCP-1 output.

**Conclusions:**

In the experimental model used, PSG had а mainly suppressive effect on the Th17 polarization and cytokine profile of Th17-polarized CD4^+^ cell cultures. As Th17 activity and a pro-inflammatory cytokine background are unfavorable during pregnancy, the observed PSG effects may play a fetoprotective role in vivo.

**Supplementary Information:**

**Supplementary information** accompanies this paper at 10.1186/s12865-020-00385-6.

## Background

During pregnancy, the maternal immune system undergoes alloimmunization by fetal antigens [1, 2]. As a result, a dynamic balance of immune tolerance formes, in which placental protein hormones are prominent [3]. Pregnancy-specific β-1-glycoproteins (PSGs) are produced during pregnancy by syncytiotrophoblast cells [4]. PSGs are the product of the expression of *psg* genes and are members of a protein family that includes more than 30 isoforms [3, 5]. The level of PSGs in peripheral blood during the third trimester reaches 200–400 μg/mL, which significantly exceeds the content of other known placental proteins [6]. PSGs are known to regulate innate and adaptive immunity [7–9]. Immunomodulatory effects of these proteins, such as the activation of TGF-β [10], stimulation of FOXP3 expression by T cells [11] and IDO expression by monocytes [12], suppression of pro-inflammatory cytokine production by intact mononuclear cells [13], and modulation of the functional activity of naive and memory T cells [14], demonstrate their important role in the formation of feto-maternal tolerance.

Among other factors, the ratio of anti-inflammatory and pro-inflammatory lymphocyte subsets (Treg and Th17) is essential for successful fetal development. During a healthy pregnancy, the Treg/Th17 ratio shifts towards Treg cells [15], and a decrease in Tregs and/or an increase in the Th17 percentage accompanies pregnancy disorders such as preeclampsia, preterm birth, misscarriage, and unexplained recurrent pregnancy loss [16]. Even in the case of a relatively successful pregnancy, an increased pro-inflammatory background may cause deviations from the typical development of the nervous system, which lead to an increased risk of neuropsychiatric disorders [17–19].

The triggering factor for these pregnancy complications may be IL-17, which induces the synthesis of the pro-inflammatory cytokines IL-1β, TNF-α, IL-6, and IL-8, along with the neutrophil-specific chemokines CXCL1, CXCL2, CXCL5, CXCL8, and RANKL; and matrix metalloproteinases (MMP-3, − 9, − 13) [20, 21].

Failed immune regulation of Th17 leads not only to pregnancy complications but also to the development of autoimmune diseases such as asthma, psoriasis, rheumatoid arthritis, Crohn’s disease, multiple sclerosis, and others [22, 23]. Interestingly, a progression of Th2-type autoimmune disease has been observed during pregnancy, while Th1/Th17-type autoimmune deseases underwent remission [24]. These data confirm that Th17-mediated immune responses are undesirable during pregnancy and are suppressed by factors that are generated during gestation.

Therefore, the objective of this study was to elucidate the effects of PSGs on the Th17 polarization of CD4^+^ cells and their cytokine production.

## Results and discussion

### The effect of PSG on the Th17 polarization of CD4^+^ cells

**Fig. 1:**
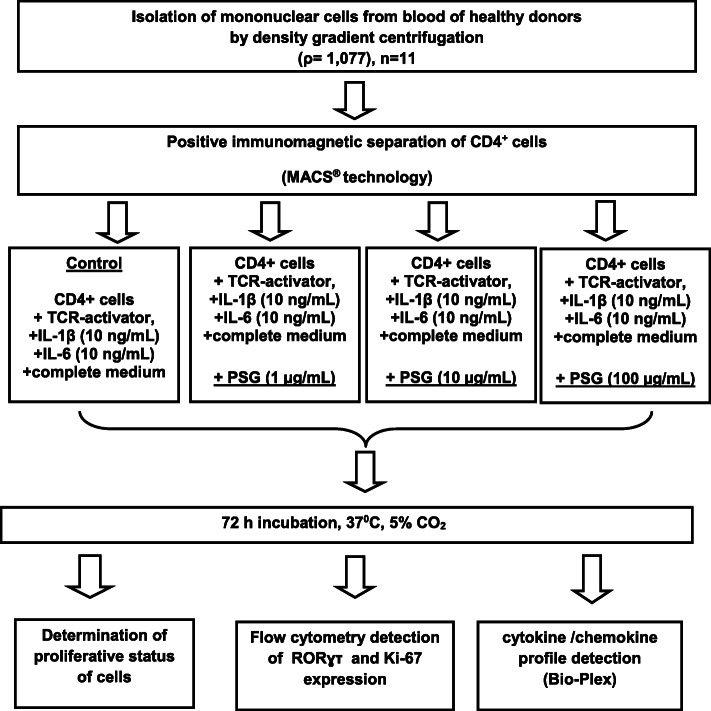
Research design

Isolated CD4^+^ T cells were cultivated for 72 h in the presence of a native PSG, TCR–activator, IL-1β, and IL-6, with subsequent assessment of proliferation using flow cytometry, detection of RORɣτ (RAR (retinoic acid receptor)-related orphan receptor gamma) and Ki-67 expression, and evaluation of different cytokine concentrations in culture supernatants (Fig. 1). We used concentrations of PSG (1, 10, and 100 μg/mL) that correspond to the first, first-second, and second-third trimesters of pregnancy, respectively [6, 25].

When estimating the effect of PSG on the proliferation of CD4^+^ T cells that accompanied their Th17 polarization, it was established that the addition of PSG into CD4^+^ cultures at concentrations of 10 and 100 μg/mL dose-dependently changed the percentages of proliferating and non-proliferating cells (Table 1). According to the differential gating method, PSG concentrations of 10 μg/mL and 100 μg/mL decreased the percentage of proliferating and increased the percentage of non-proliferating helper T cells. PSG did not affect the percentage of dead and apoptotic cells. Thus, PSG, in our study, primarily suppressed the proliferation of CD4^+^ T cells in Th17-polarizing conditions (Table 1).

**Table 1:** PSG effect on the proliferative status of CD4^+^ cells in Th17-polarizing conditions

Culture	The percentage of cells from all living lymphocytes								
	Proliferating			Non-proliferating			Apoptotic		
	M	SD	P	M	SD	P	M	SD	P
**Control**	49.91	7.718	–	47.63	7.512	–	2.458	0.331	–
**PSG 1 μg/mL**	48.99	7.935	0.7539	48.61	7.675	0.7273	2.5	0.3578	0.9672
**PSG 10 μg/mL**	47.26	6.722	**0.0258**	50.26	6.551	**0.0197**	2.479	0.2999	0.9842
**PSG 100 μg/mL**	42.34	5.642	**0.001**	55.04	5.417	**0.0011**	2.627	0.469	0.438

*n* = 11; M – arithmetic mean, SD – standard deviation, P – *P*-value; control – the CD4^+^ cell culture without PSG, but with TCR-activator, IL-1β, and IL-6. Bold *P* values indicate significant differences as compared with control by the one-way ANOVA with Dunnett’s multiple comparisons test

The antiproliferative effect of PSG was previously known [8, 26], but there was no evidence of the effect of this placental protein hormone on the Th17 pro-inflammatory subpopulation. In our previous study, the general inhibitory effect of PSG on CD4^+^ T cell proliferation was shown [27], but we now confirmed this effect for RORγτ^+^ Th17. This effect is probably due to a single mechanism of latent TGF-β1 activation for all lymphocytes [28, 29].

**Fig. 2:**
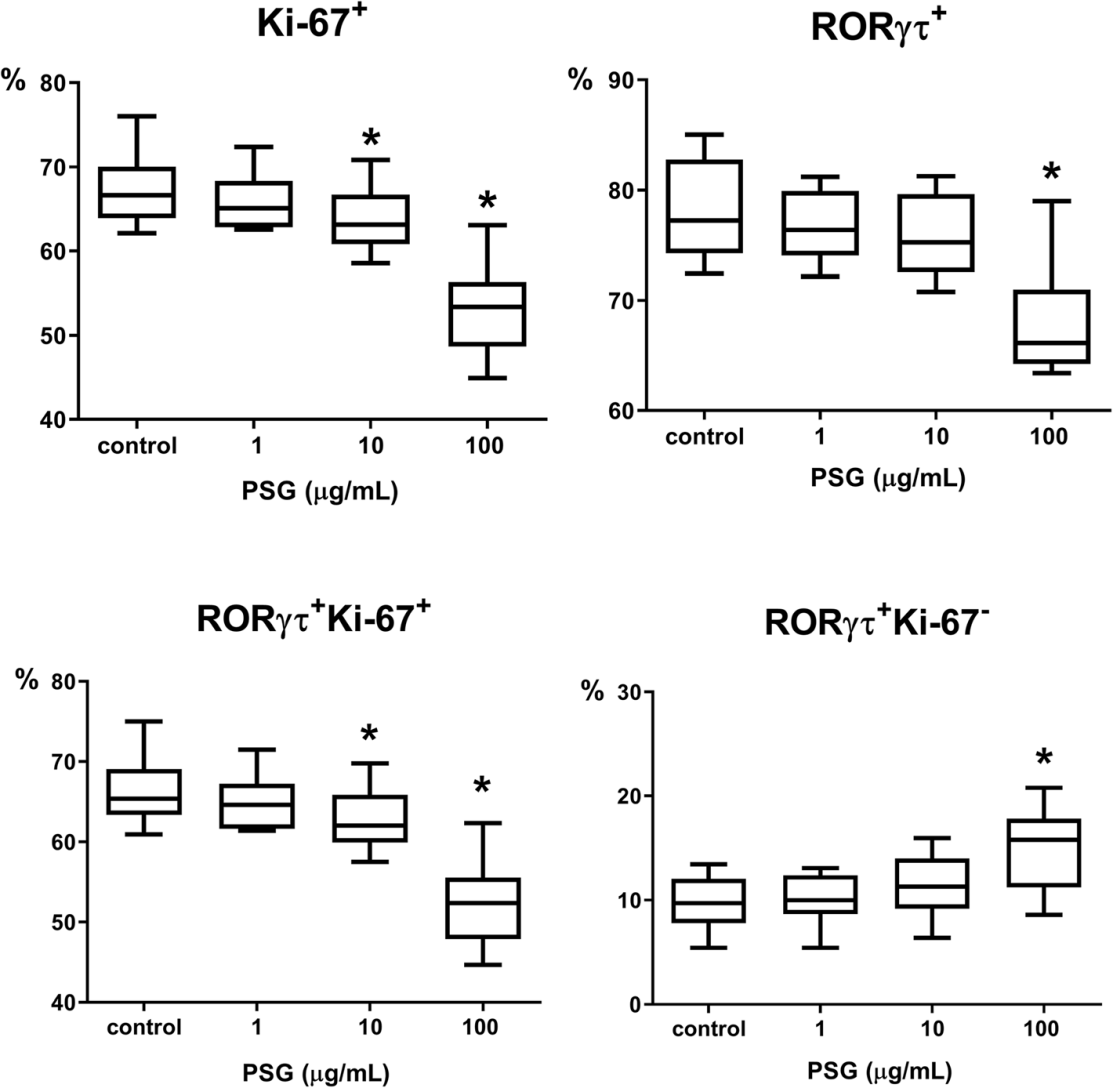
Effect of PSG on RORγτ and Ki-67 expression in the Th17-polarized CD4^+^ cell culture. Note: *n* = 11; control – the CD4^+^ cell culture without PSG, but with TCR-activator, IL-1β, and IL-6. The box shows the interquartile range (Q1-Q3), the band inside the box is the median (Me), and ends of the whiskers represent the minimum and maximum of all the data.* - significant differences (*P* < 0.05) compared with the control by the Friedman test with Dunn’s multiple comparisons test

When studying the effect of PSG on Th17 differentiation, it was found that PSG at a concentration corresponding to the last trimester of pregnancy (100 μg/mL) significantly decreased the number of CD4^+^ lymphocytes expressing the RORγτ transcription factor, the main intranuclear marker of Th17 cells [30] (Fig. 2).

The differentiation processes of Th17 cells are accompanied by the active proliferation of these cells [31]. Therefore, we evaluated the level of intracellular expression of Ki-67, which appears in actively proliferating cells [32, 33].

PSG (10 and 100 μg/mL) suppressed the expression of Ki-67 in Th17-polarized CD4^+^ cells (Fig. 2). This effect correlates with the data from the differential gating method (10 μg/mL: r = 0.69, *P* = 0.011; 100 μg/mL: r = 0.65, *P* = 0.018).

Next, we analyzed the effect of PSG on RORγτ expression, depending on the proliferative status of the cells. For this purpose, the percentages of RORγτ^+^Ki-67^+^ (proliferating) and RORγτ^+^Ki-67^−^ (non-proliferating) cells were determined (Fig. 2). PSG at concentrations of 10 and 100 μg/mL decreased the amount of proliferating Th17 (RORγτ^+^Ki-67^+^) cells in the CD4^+^ cell culture. Correspondingly, the percentage of non-proliferating Th17 cells (RORγτ^+^Ki-67^−^) in the culture increased (Fig. 2).

Thus, PSG regulates both the proliferation and differentiation of CD4^+^ cells under Th17 polarizing conditions, rendering mainly an inhibitory effect.

### The influence of PSG on the cytokine profile of Th17-polarized CD4^+^ cells

When analyzing the cytokine profile of Th17-polarized CD4^+^ cell cultures, we found that PSG at concentrations of 10 and 100 μg/mL decreased the production of IL-10, IFN-γ, MIP-1β, and TNF-α (Table 2).

**Table 2:** PSG effect on the cytokine/chemokine profile of the Th17-polarized CD4^+^ cell culture

Cytokine (pg/mL)	Control	PSG concentration		
		1 μg/mL	10 μg/mL	100 μg/mL
**IL-2**	335,63 (245,64-442,40)	291,51 (205,12-614,36)	283,09 (196,76-597,03)	245,33 (220,51-360,54)
**IL-4**	11,7 (10,66-13,06)	11,26 (8,98-12,65)	10,10 (8,79-11,36)	10,46 *(9,21-11,21)
**IL-5**	275,23 (255,28-292,34)	281,69 (231,28-294,73)	240,85 (231,14-281,29)	226,75 *(215,79-245,03)
**IL-7**	2,44 (2,08-2,63)	1,73 (1,57-2,73)	2,51 (1,61-2,81)	1,73 (1,61-1,79)
**IL-8**	2568 (1774–3204)	1748,39 (1084,57–2207,15)	1632,86 (1512,27-3047,92)	1807,68 *(1109,83-2386,00)
**IL-10**	1125,84 (968,65-1605,55)	728,61 (387,41-1330,60)	634,33 *(576,84-843,67)	321,45 *(255,58-745,42)
**IL-12**	7,18 (6,19-8,15)	6,71 (6,28-7,95)	6,81 (6,17-8,52)	5,80 *(5,70-7,11)
**IL-13**	360,56 (301,15-506,65)	271,43 (238,28–774,10)	252,04 (185,75-305,85)	165,30 *(109,11-295,97)
**IL-17**	7836 (6093–8037)	7873,86 (1328,34-8082,78)	3239,92 (1611,37-7955,67)	2966,21 *(1280,53-5110,84)
**G-CSF**	35,45 (25,44-42,35)	24,52 (19,87-28,87)	22,24 (17,49-31,48)	19,39 *(16,79-23,40)
**GM-CSF**	651,87 (524,99-786,04)	432,00 (260,92-653,92)	321,18 (186,05-508,75)	237,85 *(67,11-275,20)
**IFN-ɣ**	987,56 (746,74-1142,26)	764,75 (605,65-1125,11)	609,74 *(417,27-693,11)	381,27 *(236,37-726,22)
**MCP-1**	39,62 (37,53-43,76)	37,86 (32,58-40,98)	36,87 (26,57-43,23)	35,46 (30,97-45,22)
**MIP-1β**	1844,29 (1702,16-1878,91)	1851,78 (980,31-1891,95)	1177,67 *(794,94-1827,56)	884,21 *(694,04-1572,02)
**TNF-α**	7357,46 (6516,69-14,856,20)	4349,65 (2888,17-9145,45)	3908,57 *(2726,18-6163,68)	3494,69 *(2965,56-4781,27)

*n* = 11; Medians with interquartile range (Me(Q1-Q3)) are presented. Control – the CD4^+^ cell culture without PSG, but with TCR-activator, IL-1β, and IL-6. * - Significant differences (*P <* 0.05) compared with the control by the Friedman test with Dunn’s multiple comparisons test

In some cases, an inhibitory effect of only a high concentration of PSG on the production of cytokines was found. PSG at concentration of 100 μg/mL suppressed the production of IL-4, IL-5, IL-8, IL-12, IL-13, IL-17, G-CSF, and GM-CSF (Table 2). Among the above-mentioned cytokines, the pregnancy protein had the most pronounced inhibitory effect on the secretion of IL-17 (2.64-fold) and GM-CSF (2.74-fold).

Reduction in IL-17 output in 100 μg/mL PSG CD4^+^ culture correlates with a decrease in the proliferating RORγτ^+^Ki67^+^ cell percentage and inversely correlates with an increase in the non-proliferating RORγτ^+^Ki67^−^ cell percentage and thus may be directly associated with the antiproliferative effect of PSG.

In addition to IL-17, their major effector cytokine, Th17 cells can produce IL-21, IL-22, TNFα, IFNγ, and GM-CSF. This impressive arsenal helps cells cope with extracellular and intracellular bacteria; ensure protective immunity to *Mycobacterium tuberculosis, Chlamydia trachomatis*, fungi, and viruses; protect mucosal homeostasis; and enhance the neutrophil response. Conversely, it is involved in inflammation and several autoimmune diseases [34]. IL-17 has been shown to activate the nuclear factor (NF)-kB downstream signaling pathway, which results in the expression of pro-inflammatory cytokine genes, such as TNF-α, IL-1, IL-6, G-CSF, and GM-CSF; the chemokines CXCL1, CXCL5, IL-8, CCL2, and CCL7; the matrix metalloproteinases MMP1, MMP3, MMP9, and MMP13; and the antimicrobial peptides defensins and S100 proteins [35]. Since IL-17 is a decisive factor triggering inflammatory reactions, it is quite logical that in our study, a decrease in its concentration in 100 μg/ml PSG cultures correlated with a decrease in TNF-α (r = 0.78, *P* = 0.003) and IFN-γ (r = 0.73, *P* = 0.007). TNF-α, like other IL-17-derived pro-inflammatory cytokines, is highly undesirable during pregnancy, as it can causе pregnancy complications, such as recurrent miscarriage, premature rupture of fetal membranes, preeclampsia and intrauterine fetal growth retardation [36].

In our study, PSG at a concentration of 100 μg/mL inhibited the production of G-CSF and GM-CSF by CD4^+^ cells under Th17-polarizing conditions (Table 2). These hematopoietic colony-stimulating factors are necessary for the onset and progression of pregnancy, but above all, locally. Other pregnancy proteins may stimulate their synthesis in vivo. In particular, it is known that the expression of GM-CSF is triggered by chorionic gonadotropin [37], the concentration and therefore the dose-dependent effect of which are significantly higher in the first trimester of pregnancy than PSG.

In addition to inhibiting the “classic” pro-inflammatory cytokines and chemokines, PSG, in our study, also decreased the production of anti-inflammatory cytokines such as Il-4, IL-10, and IL-13. The decrease in IL-10 output in the 10 μg/mL PSG culture was inversely correlated with the percentage of RORγτ^+^ cells. This result is logical since the main producers of IL-10 are Treg, Th2, and the effector Th1 [34, 38]. The presence of these cells in the cultures is very likely since we Th17 polarized not only naive but all CD4^+^ T cells. The cultures initially contained a mixture of different T helper subsets, including Th1, T reg, and Th17, which are known to have plasticity and can transdifferentiate from one subpopulation to another, depending on the cytokine environment [39, 40]. Most likely, the main portion of helper T cells polarized into RORγτ^+^ Th17 due to the cytokine background (Fig. 2); however, some of the CD4^+^ T cells “remained true” to their original phenotype.

Interestingly, although the concentration of IL-4 in cultures of Th17-polarized CD4^+^ cells was initially low in the control (Me = 11.7 pg/ml), in the 100 μg/mL PSG culture, there was a significant decrease in the production of this cytokine (Me = 10.46 pg/ml), which correlated (r = 0.67, *P* = 0.014) with a reduction in the percentage of Ki67^+^ and RORγτ^+^Ki67^+^ cells. Perhaps, due to the plasticity of the T helper phenotypes, a small percentage of transitional cell subsets co-expressing, along with IL-17, a variety of other cytokines, including IFN-γ, IL-10, and IL-4, is formed in the pro-inflammatory cytokine environment, as occurs with autoimmune diseases [40].

Regarding the suppression of cytokine production, our previous studies have already shown a simultaneous decrease in the production of both pro-inflammatory (IFN-γ) and anti-inflammatory (IL-4) cytokines [27] by CD4^+^ T cells under the influence of PSG. This effect can probably be associated with the “universal” antiproliferative effect of this pregnancy hormone since, in this study, a correlation of the percentage of Ki67^+^ (proliferating) cells was detected with the decrease in concentrations of several cytokines, including IL-4, IL-17, GM-CSF, IFN-γ, and TNF-α, and the chemokine MIP-1β [28, 29, 41].

## Conclusions

Thus, in the experimental model used, PSG had an expressed suppressive effect on the proliferation and Th17 polarization of CD4^+^ T cells and on their cytokine/chemokine production. As Th17 activity and an increase in pro-inflammatory cytokine production are unfavorable during pregnancy, the revealed PSG effects may play a fetoprotective role in vivo.

## Materials and methods

### Study groups

Venous blood samples were collected from healthy donors (non-pregnant women, *n* = 11, 21–39 years old) by venipuncture with vacuum tubes (BD Vacutainer™, Greiner-bio-one, Austria).

Peripheral blood mononuclear cells (PBMCs) were isolated by density gradient centrifugation (Diacoll 1077, Dia-m, Russia, ρ = 1.077 g/cm^3^). The maximum time between collection of a blood sample and separation in a density gradient was 30 min.

### Native PSG isolation

PSG was purified from the blood of healthy pregnant women according to the technique developed by Mikhail Rayev [42] and described in detail elsewhere [27]. PSG preparation is a mixture of the following proteins: PSG1, PSG3, PSG7, PSG9, and some of their isoforms and precursors.

### Isolation and cultivation of CD4^+^ cells

CD4^+^ cells were obtained from PBMCs by positive immunomagnetic separation (MACS® MicroBeads and MS Columns, Miltenyi Biotec, Germany). The purity of the isolated T helper population was checked using a two-color BD Simultest™ IMK-Lymphocyte kit (Becton, Dickinson and Company, BD Biosciences, USA) and a CytoFLEX S Flow Cytometer (Beckman Coulter, USA). The percentage of B-cells (CD3^−^CD19^+^) did not exceed 0.02%, monocytes (CD45^+^CD14^+^) – 0.2%, CTLs (CD3^+^CD8^+^) – 1%, and NK-cells (CD3^−^CD16/56^+^) – 0.05%. The portion of CD3^+^CD4^+^ cells varied within 97–99%.

Isolated CD4^+^ cells were cultured in 96-well plates (1 × 10^6^ cells/mL, 200 μL) in complete medium (CM), RPMI-1640 (Sigma-Aldrich, USA) supplemented with 10% FBS (Sigma, USA), 10 mM HEPES, 2 mM L-glutamine (both from ICN Pharmaceuticals, USA), and 30 μg/mL gentamycin (KRKA, Slovenia), and in a humidified CO_2_ incubator at 37 °C and 5% CO_2_ for 72 h.

Cultures without PSG served as a control. The viability of the cells after incubation evaluated using 0.4% trypan blue (Invitrogen, USA) was 95–98%. PSG did not affect either the cell number or the viability of cells.

### Th17 polarization

To polarize CD4^+^ cells into Th17 cells, we used TCR-activator (T Cell Activation/Expansion Kit human, Miltenyi Biotec, Germany) and the IL-1β and IL-6 cytokines at concentrations of 10 ng/mL (Miltenyi Biotec, Germany) [43].

Th17 polarization resulted in a significant increase in the percentage of proliferating CD4^+^ cells (Me (Q1-Q3), from 11.32 (9.32–15.63) to 42.33 (34.03–50.14)), accompanied by a simultaneous decrease in the percentage of non-proliferating cells (from 83.56 (77.85–97.53) to 47.84 (39.47–53.57)), while the level of apoptosis cells did not change. In general, this result indicates an adequate activation of CD4^+^ cells in the present experimental model. The obtained data are consistent with similar experiments, where we studied the proliferation of TCR-activated helper T cells in the presence of IL-2 [44].

The independent effect of Th17 polarization consisted of a significant (more than 60-fold) increase in the number of cells expressing Ki-67 (Additional file 1, Table S1).

The effect of Th17 polarization on the cytokine/chemokine profile of CD4^+^ cells was expressed as a reliable increase in IL-2, IL-4, IL-5, IL-7, IL-10, IL-17, G-CSF, GM-CSF, IFN-γ, MIP-1β, and TNF-α output. The most pronounced increase was observed in the TNF-α and IL-17 concentrations, thus confirming the predominance of IL-17-producing cells in culture and, in general, the successful formation of a pro-inflammatory background (Additional file 2, Fig. S1).

### Flow cytometry

The main transcription factor of Th17 cells is RORγτ [26]. Therefore, after 72 h of cultivation, we determined the frequency of Th17 cells as a percentage of RORγτ^+^ CD4^+^ cells. To evaluate the percentage of proliferating Th17 cells, we assessed the level of intracellular Ki-67 protein.

Sample preparation for intracellular/intranuclear staining was performed with a FOXP3 Fix/Perm Buffer Set (Biolegend, USA). Stained samples were analyzed by running a two-color flow cytometry assay with a CytoFLEX S (Beckman Coulter, USA). The antibodies used were anti-RORg(t)-PE, human and mouse, and anti-Ki-67-PerCP-Vio700™, human and mouse (both Miltenyi Biotec, Germany).

The frequencies of RORγτ^+^, Ki-67^+^, ROR-γτ^+^Ki-67^+^, and RORγτ^+^Ki-67^−^ cells were assessed. The data are presented as percentages of RORγτ^+^, Ki-67^+^, RORγτ^+^Ki-67^+^, and RORγτ^+^Ki-67^−^ cells from all events in the gate of living СD4^+^ lymphocytes established according to FSC and SSC properties. The threshold between positive and negative cells was determined using the fluorescence minus one (FMO) controls. Flow cytometry data were analyzed using CytExpert 2.0 software (Beckman Coulter, USA).

### Proliferation analysis

An author-modified differential gating method [45] was used to determine the proliferative status of cells. In our study, in contrast to the above-referenced method, we did not calculate the absolute but rather the relative number of proliferating cells. That is, we calculated the percentage of cells in each gate (proliferating, non-proliferating, and apoptotic) from all cells in the three gates [27, 46].

Data were acquired on a CytoFLEX S Flow Cytometer and analyzed in CytExpert 2.0 software (Beckman Coulter, USA).

### Evaluation of the cytokine profile

After 72 h of incubation in the culture supernatants of Th17-polarized CD4^+^ cells, the IL-2, IL-4, IL-5, IL-7, IL-8, IL-10, IL-17, G-CSF, GM-CSF, IFN-γ, MCP-1, MIP-1β, and TNF-α concentrations were determined in the supernatants of CD4^+^ cell cultures using a Bio-Plex® Multiplex Immunoassay System (Bio-Rad, USA).

Procedures were performed according to the “Bio-Plex Pro™ Human Cytokine 27-plex Assay” and “Bio-Plex Pro™ Human Th17 Cytokine Panel 15-Plex” protocols. The results were recorded using a Bio-Plex automatic microplate photometer and Bio-Plex Manager software (Bio-Plex® 200 Systems, Bio-Rad, USA). The cytokine concentrations were determined from a calibration curve for each cytokine (dynamic range from 2 to 32,000 pg/mL) according to the manufacturer’s recommendations.

### Statistics

The **s**tatistical data analysis was performed with GraphPad Prism 6 using one-way ANOVA (proliferation, differential gating) and the paired Friedman test with Dunn’s multiple comparisons test (RORɣτ and Ki-67, cytokines). Data are presented as arithmetic means and standard deviations (M ± SD) and medians with first and third quartile values (Me (Q1 − Q3)), respectively. Differences were considered significant at *P* < 0.05. In some cases, the Spearman correlation coefficient was calculated.

## Supplementary Information

**Additional file 1: Table S1**. The effect of Th17 polarization on the RORγτ and Ki-67 expression in CD4^+^ T cells. Medians and interquartile ranges of cell percentages from all alive CD4^+^ T-cells are presented (Me (Q1–Q3)). Control – CD4 ^+^ cell culture with CM only, * - Significant differences (*P* < 0.05) compared with the control by the one-way ANOVA with Dunnett’s multiple comparisons test; *n* = 11.

**Additional file 2: Fig. S1**. Cytokine profile of Th17-polarized CD4^+^ cell culture. Medians of cytokine concentrations in control CD4^+^ culture (with TCR-activator, IL-1β, and IL-6) are presented; *n =* 11. The box shows the interquartile range (Q1-Q3), the band inside the box is the median (Me), and the ends of the whiskers represent the minimum and maximum of all the data.

## Data Availability

The datasets of flow cytometry data generated during the current study are available at FlowRepository, http://flowrepository.org/id/FR-FCM-Z2HS.

## Abbreviations

PSG: Pregnancy-specific β1-glycoprotein
Th17: IL17-producing helper T cells
TCR: T-cell receptor
CM: Complete medium
