# Supplementary material for: The effects of human pregnancy-specific β1-glycoprotein preparation on Th17 polarization of CD4+ cells and their cytokine profile
Source: BMC Immunol. 2020 Oct 30;21:56. doi: 10.1186/s12865-020-00385-6 (PMC7602336; doi:10.1186/s12865-020-00385-6)
Supplement: Supplementary file 1 — Additional file 1: Table S1. The effect of Th17 polarization on the RORγτ and Ki-67 expression in CD4+ T cells. Medians and interquartile ranges of cell percentages from all alive CD4+ T-cells are presented (Me (Q1–Q3)). Control – CD4 + cell culture with CM only, * - Significant differences (P < 0.05) compared with the control by the one-way ANOVA with Dunnett’s multiple comparisons test; n = 11. [file 12865_2020_385_MOESM1_ESM.docx]

**Table S1**

**The effect of Th17 polarization on the RORγτ and Ki-67 expression in CD4^+^ T cells**

|  | RORγτ^+^  % | Ki-67^+^  % | RORγτ^+^Ki-67^-^  % | RORγτ^+^Ki-67^+^  % |
| --- | --- | --- | --- | --- |
| Control | 12.77  (10.23–16.54) | 0.93  (0.26–1.76) | 11.74  (6.13-20.43) | 0.43  (0.11–0.98) |
| Control  + TCR-activator  + IL-1β, IL-6 | 77.25 *  (73.73-83.48) | 64.20 *  (47.45-68.10) | 11.83  (8.71-22.82) | 63.53*  (47.13-66.64) |

*Note: n=11; median and interquartile range of cell percentages from all alive CD4^+^ T-cells* are presented (Me (Q1–Q3)). *Control – CD4^+^cell culture with CM only, * - Significant differences (P<0.05) compared with the control by the one-way ANOVA with Dunnett's multiple comparisons test.*
