# Supplementary material for: The effects of human pregnancy-specific β1-glycoprotein preparation on Th17 polarization of CD4+ cells and their cytokine profile
Source: BMC Immunol. 2020 Oct 30;21:56. doi: 10.1186/s12865-020-00385-6 (PMC7602336; doi:10.1186/s12865-020-00385-6)
Supplement: Supplementary file 2 — Additional file 2: Fig. S1. Cytokine profile of Th17-polarized CD4+ cell culture. Medians of cytokine concentrations in control CD4+ culture (with TCR-activator, IL-1β, and IL-6) are presented; n = 11. The box shows the interquartile range (Q1-Q3), the band inside the box is the median (Me), and the ends of the whiskers represent the minimum and maximum of all the data. [file 12865_2020_385_MOESM2_ESM.docx]

**

**

**Fig. S1. Cytokine profile of Th17-polarized CD4^+^ cell culture.** *Note:* *n=11;* *medians of cytokine concentrations in control CD4^+^ culture (with TCR-activator, IL-1β, and IL-6) are presented*. *The box shows the interquartile range (Q1-Q3), the band inside the box is* *the median (Me), and ends* *of the whiskers represent the minimum and maximum of all the data.*
